# Spatial genetic structure in a crustacean herbivore highlights the need for local considerations in Baltic Sea biodiversity management

**DOI:** 10.1111/eva.12914

**Published:** 2020-02-05

**Authors:** Pierre De Wit, Per R. Jonsson, Ricardo T. Pereyra, Marina Panova, Carl André, Kerstin Johannesson

**Affiliations:** ^1^ Department of Marine Sciences University of Gothenburg Tjärnö Sweden; ^2^ Environmental and Marine Biology Åbo Akademi University Turku Finland

**Keywords:** Baltic Sea, connectivity, *Idotea balthica*, marine protected areas, seascape genetics

## Abstract

Incorporating species' eco‐evolutionary responses to human‐caused disturbances remains a challenge in marine management efforts. A prerequisite is knowledge of geographic structure and scale of genetic diversity and connectivity—the so‐called seascape genetic patterns. The Baltic Sea is an excellent model system for studies linking seascape genetics with effects of anthropogenic stress. However, seascape genetic patterns in this area are only described for a few species and are completely unknown for invertebrate herbivores, which constitute a critical part of the ecosystem. This information is crucial for sustainable management, particularly under future scenarios of rapid environmental change. Here, we investigate the population genetic structure among 31 locations throughout the Baltic Sea, of which 45% were located in marine protected areas, in one of the most important herbivores of this region, the isopod crustacean *Idotea balthica*, using an array of 33,774 genome‐wide SNP markers derived from 2b‐RAD sequencing. In addition, we generate a biophysical connectivity matrix for *I. balthica* from a combination of oceanographic current models and estimated life history traits. We find population structure on scales of hundreds of kilometers across the Baltic Sea, where genomic patterns in most cases closely match biophysical connectivity, indicating passive transport with oceanographic currents as an important mean of dispersal in this species. We also find a reduced genetic diversity in terms of heterozygosity along the main salinity gradient of the Baltic Sea, suggesting periods of low population size. Our results provide crucial information for the management of a key ecosystem species under expected changes in temperature and salinity following global climate change in a marine coastal area.

## 1 INTRODUCTION

The capacity of a species to disperse across the land/seascape is one of the most important traits determining its resilience to environmental change (Travis et al., 2013). Dispersal counteracts population fragmentation, which tends to render more isolated populations vulnerable to local stressors such as over‐exploitation or habitat degradation (Doerr et al. 2011). EDConnectivity, dispersal behaviour and conservation under climate change: A response to Hodgson. In addition, effects of large‐scale gradual changes such as climate change are less profound in species with high dispersal capabilities, as they are better able to shift their distribution ranges through migration (Doerr et al., 2011). Species with poor dispersal abilities living in areas with physical barriers to dispersal are particularly vulnerable, so knowledge about where these barriers are located, and in which areas isolated populations are found, is two pieces of information critical to management efforts worldwide (Selkoe et al., 2016). In the marine environment, the realized gene flow resulting from various types of larval/propagule dispersal, as well as juvenile and adult movements, has for long remained unknown for a majority of species. The apparent lack of obvious dispersal barriers in the oceans led earlier biologists to conclude that marine populations show little fragmentation. However, a multitude of studies have later shown that many marine species are spatially structured on smaller scales than previously thought (reviewed in Selkoe et al., 2016). In order to better understand the location of dispersal barriers and their consequences, seascape genetic studies are performed by connecting biophysical dispersal models, ecology, and population genetic data (Galindo, Olson, & Palumbi, 2006). This information is highly relevant for managers in conservation of species, for example, in the establishment and/or evaluation of networks of marine protected areas (MPAs) (Underwood, Smith, Van, & Gilmour, 2009). Further, future species distributions will shift as a consequence of climate change, with important management implications. For example, Loarie et al. (2009) showed that due to climate change, on a global scale only 8% of protected areas designed today would still contain the habitat they were designed to protect in 100 years from now. In coastal areas, one strategy for mitigation would be to ensure that geographic areas likely to be strongly impacted by the changing environment are well connected to other suitable areas through dispersal corridors (Jonsson, Kotta, & Andersson, 2018), where one key feature would be protection of habitat‐forming species.

**Figure 1:**
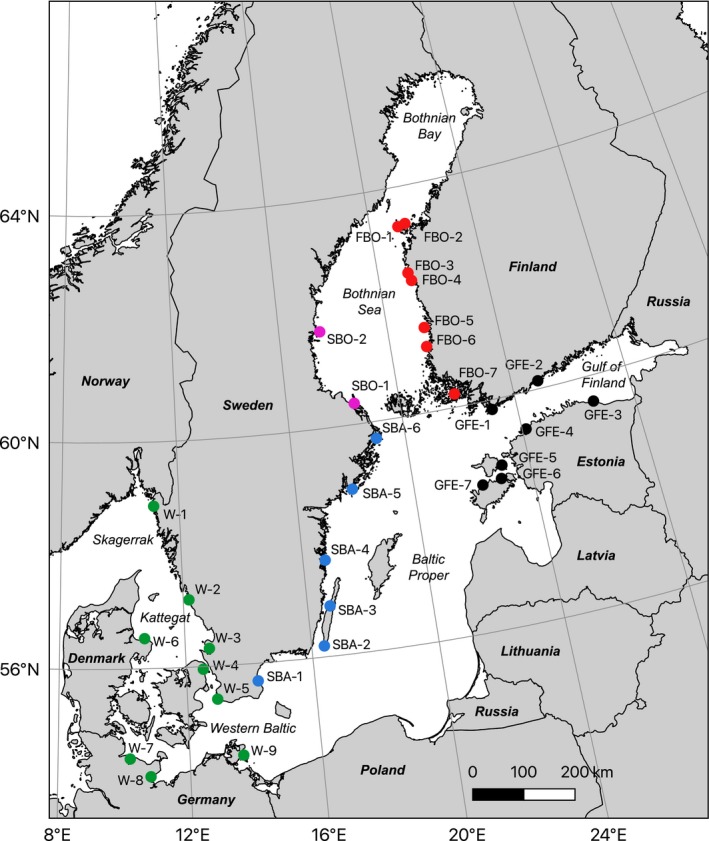
Map of the study area (the Baltic Sea), with collecting locations marked with dots colored by region

The Baltic Sea (Figure 1) is an ideal area to study the interaction between biophysical connectivity, eco‐evolutionary dynamics, and population genetic structure. This is primarily because oceanographic patterns are relatively well described in the Baltic Sea (Hordoir et al., 2019). The Baltic Sea is a multibasin marginal sea, with rather strong oceanographic barriers and sharp environmental gradients separating the different basins (Leppäranta & Myrberg, 2009). There is also limited oceanographic connectivity between the Baltic and the adjacent North Sea (Moksnes, Corell, Tryman, Hordoir, & Jonsson, 2014), effectively isolating Baltic populations of marine organisms and reducing gene flow among coastal populations of marine species. Furthermore, the connection to the North Sea opened recently, about 8,000 years ago (Ignatius, Axberg, Niemistö, & Winterhalter, 1981), and while the majority of marine species in the Atlantic failed to colonize it completely, others have reached differently far into the Baltic Sea and along its strong salinity gradient (Ojaveer et al., 2010). These distributional differences may be due to, for example, differences in stress tolerance (Weinberger, Buchholz, Karez, & Wahl, 2008; Wrange et al., 2014), dispersal (Sjöqvist, Godhe, Jonsson, Sundqvist, & Kremp, 2015; Urho, 1999), or reproductive abilities across species (Jaspers, Møller, & Kiørboe, 2011). In addition, what seems common to many of these marine species is a strong genetic divergence between the North Sea and Baltic Sea populations, with a sharp boundary at the North Sea–Baltic Sea environmental transition zone, which is located just southeast of the Danish straits (Johannesson & André, 2006). There are multiple potential reasons for the maintenance of this boundary, including local adaptation to the steep salinity cline in the area in combination with a reduced connectivity into the Baltic Sea by current patterns (Le Moan, Jiménez‐Mena, Bekkevold, & Hemmer‐Hansen, 2019a). Also, reproductive incompatibilities arisen from historical geographic separation, in some cases before the opening of the Baltic Sea, could also act to maintain population separation in the face of secondary contact (Bierne, Welch, Loire, Bonhomme, & David, 2011; Le Moan, Gaggiotti, Henriques, & Martinez, 2019b; Le Moan, Jiménez‐Mena, et al., 2019a).

The Baltic Sea has recently been proposed as a reference and model system (a "time‐machine") for other coastal areas as it is ahead of other areas with respect to issues such as impacts of climate change, habitat loss, eutrophication, pollution, and over‐fishing, as well as being at the forefront when it comes to both monitoring, scientific investigations, and management measures (Reusch et al., 2018). The Baltic Sea is one of the fastest‐warming regions in the world (Reusch et al., 2018), which will be accompanied by additional increased precipitation resulting in a strong reduction in salinity (Meier, Hordoir, et al., 2012a). This is expected to lead to substantial range shifts of species with loss of marine species, expansion of freshwater species, and continued rapid introduction of new species (Ojaveer & Kotta, 2015). For management of individual species, knowledge of dispersal barriers and population fragmentation is critical to improve measures. One major concern is whether northern Baltic Sea populations possess dispersal capabilities and adaptability to higher temperatures, or to salinity reductions and shift their ranges to the south to escape extinction (Johannesson, Smolarz, Grahn, & Andre, 2011; Jonsson et al., 2018).

Seascape genetic studies are scarce in the Baltic Sea. The studies undertaken to date focus mainly on commercially caught fish species (Östman, Olsson, Dannewitz, Palm, & Florin, 2017; Wennerström, Jansson, & Laikre, 2017), which show highly species‐specific patterns. Some fish, such as perch and herring, are clearly structured geographically (Lamichhaney et al., 2012; Olsson, Mo, Florin, Aho, & Ryman, 2011; Teacher, André, Jonsson, & Merilä, 2013), as are species with distinct spawning areas such as cod and salmon (Barth et al., 2019; Berg et al., 2015; Poćwierz‐Kotus et al., 2015). In addition, a few studies using a restricted set of genetic markers on the brown algal habitat‐founding species *Fucus vesiculosus* and *Fucus radicans* (Ardehed et al., 2016; Pereyra et al., 2013) found very distinct population structure patterns. However, in different species of flatfish this does not seem to be the case (Le Moan, Gaggiotti, et al., 2019b; Nielsen, Nielsen, Meldrup, & Hansen, 2004), highlighting that population genetic patterns often are species‐specific and depend on traits such as dispersal potential and population sizes.

One missing key piece of the puzzle of seascape genetics in the Baltic Sea is the mesograzer herbivore guild—small, mostly crustacean grazers, which use macroalgae for food and shelter and in turn provide an important food source for fish. Three species of isopods of the genus *Idotea* are arguably the most important herbivores in the depauperate Baltic Sea ecosystem (Leidenberger, Hårding, & Jonsson, 2012), the most common one being *Idotea balthica*. They are generalist grazers on different algae and sea grasses, but also have the uncommon ability to survive and grow solely on a diet of brown algae of the genus *Fucus* (Bell & Sotka, 2012). Indeed, in some areas in the Baltic, densities of *I. balthica* can rise to astonishing numbers (more than 80 individuals/100 g wet weight of *Fucus*) and their grazing can severely decimate *Fucus* in a local area (Engkvist, Malm, & Tobiasson, 2000). While these invertebrates thus form an important ecological function, no previous studies have examined population genetic patterns or dispersal potentials of these species in the Baltic Sea. *Idotea* species brood their young and have no pelagic larval dispersal phase, which would largely prevent dispersal. On the other hand, adults are strong swimmers, and in addition, they have the potential to raft long distances and cross open‐sea barriers attached to free‐floating algae (Rothausler, Corell, & Jormalainen, 2015; Thiel & Gutow, 2005), which might counteract the lack of larval dispersal.

To investigate the dispersal potential of mesograzers in the Baltic Sea, we here use a seascape genetics approach applied on *I. balthica*. Our objectives are to describe the population genetic structure and identify barriers to dispersal in the Baltic Sea region. We also aim to locate hotspots of genetic diversity, which could be considered to be of higher protective value, as well as isolated areas of low diversity and low connectivity which might be at higher risk of local extinction. In order to achieve these goals, we combine biophysical transport models and population genomic data of high spatial resolution and test for concordance between genetic and biophysical model data. If patterns are concordant, it will be possible to extrapolate genetic patterns geographically in order to generate maps that could be used in management efforts. Thus, the information produced here provides important input for future considerations in the Baltic Sea MPA network.

We collected isopods from outside of the entrance of the Baltic Sea to the innermost areas of the species' distribution range (the Bothnian Sea and the Gulf of Finland), with almost half of the sites being MPA sites. Using 2b‐RAD sequencing (Wang, Meyer, McKay, & Matz, 2012), we identified approximately 35,000 SNP markers randomly distributed across the genome, which we used to estimate population differentiation, barriers to dispersal, and areas of high and low genetic diversity. We also estimated a connectivity matrix using a biophysical model based on oceanographic circulation patterns combined with hypothesized dispersal traits of *I. balthica*, which we compared to the population genetic structure indicated by the genomic data. We hypothesized that the lack of larval dispersal in this species has resulted in strong population fragmentation along the Baltic Sea coast and that the population genetic structure follows an isolation‐by‐distance pattern where oceanographic connectivity largely explains the genetic patterns. We explicitly compared genetic diversity within and outside of HELCOM MPA sites. According to international conventions, placement of MPA sites should take genetic diversity into account (e.g., EU Framework Directive 2008/56/EC). If the current marine MPA network within the study area has been optimally designed in this respect, we would expect samples from MPAs to contain higher genetic diversity than non‐MPA ones. Further, we hypothesize that the *I. balthica* population in the Baltic Sea has a reduced genetic diversity compared to outside locations, due to periodically low population sizes either before, during, or after the colonization of the Baltic, as has been observed in other species (Johannesson & André, 2006).

## 2 MATERIALS AND METHODS

### 2.1 Field collection

Individuals of *Idotea balthica* were collected from 31 locations in the Baltic Sea (including Kattegat/Skagerrak) by snorkeling and picking isopods from *Fucus* from August 8 to October 2, 2014 (except/W‐1, which was sampled on May 15, 2015), with 14 locations being within HELCOM MPAs (Table 1). From hereon, a group of isopod individuals collected at one specific sampling location and time will be referred to as a *sample*. Animals were decapitated and stored in 95% ethanol at −20°C. From most locations, DNA from 20 individuals was extracted, four of which twice for technical replication, using a Qiagen Blood & Tissue kit and following the standard protocol, with the addition of RNAse during the last 30 min of the tissue lysis step in order to avoid RNA contamination. DNA quantity was measured using a QuBit dsDNA BR assay, and quality was assessed through gel electrophoresis.

**Table 1:**
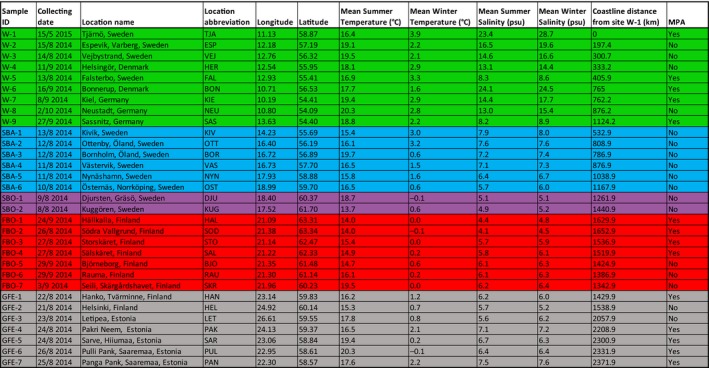
Collecting location information. Broad‐scale geographic regions are color‐coded as follows: Green—Western Baltic Sea (Skagerrak/Kattegatt/Danish Straits area) (W); Blue—Swedish Baltic Sea proper (SBA); Purple—Swedish Bothnian Sea (SBO); Red—Finnish Bothnian Sea (including the Archipelago Sea) (FBO); Black—Gulf of Finland and Estonia (GFE). Color codings are used consistently throughout the manuscript. Summer and winter values for temperature and salinity are averages 1995–2004 for June–August and December–February, respectively

### 2.2 Genotyping

2b‐RAD libraries (Wang et al., 2012) were prepared from the DNA using a modified version of the laboratory protocol developed by Mikhail Matz, available at: https://github.com/DeWitP/BONUS_BAMBI_IDOTEA. In brief, 100–200 ng of DNA template was fragmented using the type 2b endonuclease enzyme BcgI, after which adapters were ligated to the ends of the excised 36‐bp fragments. Fragments were then amplified with barcoded adapters, after which they were pooled equimolarly into 24‐sample population pools (20 individuals + 4 technical replicates). All pools were sequenced in an Illumina HiSeq 2,500 machine, 50bp single‐end, at the Swedish National Genomics Infrastructure's SNP & SEQ platform at Uppsala University.

All bioinformatic analyses were run on the University of Gothenburg computer cluster “Albiorix” (http://albiorix.bioenv.gu.se/). All commands used in the analyses can be found here: https://github.com/DeWitP/BONUS_BAMBI_IDOTEA. An unpublished draft genome assembly for *I. balthica* was used as a reference for mapping the 2b‐RAD data, using bowtie2 (Langmead & Salzberg, 2012) (information for the genome project can be found at https://github.com/The-Bioinformatics-Group/Idotea_genome_project). The standard GATK pipeline (McKenna et al., 2010) was followed for SNP calling (using the UnifiedGenotyper), including InDel realignment, quality score recalibration (BQSR), and variant score quality recalibration (VQSR), using sites identically genotyped across all technical replicates as a “True” training set for the machine learning algorithm. Poorly genotyped individuals and SNP sites genotyped at < 80% of all individuals were filtered out, as well as highly (>75%) heterozygous sites. The data set was pruned in order to keep only one SNP site for each RAD fragment. Finally, technical replicates examined for concordance in genotype estimates were discarded from further analysis.

### 2.3 Among‐sample summary statistics and differentiation

Mean pairwise *F*_ST_, as well as sample allele frequency differentiation p‐values using Fisher's exact probability test, was calculated for all loci combined for all sample pairs using GENEPOP (Raymond & Rousset, 1995; Rousset, 2008) (Option 3, “Genic differentiation for all pairs of populations,” with default Markov chain parameters). This method computes sample allele frequencies and seeks to reject the null hypothesis that alleles in both samples are drawn from the same distribution. Pairwise *F*_ST_ values were transformed using a metric multidimensional scaling (MDS) approach, which summarizes the genetic distances in a multidimensional plane with as few dimensions as possible, after which the first two dimensions were plotted using R. Genetic diversity indices (*F*_IS_ and expected heterozygosity *H*_E_) were calculated using Arlequin v.3.5.2.2 (Excoffier & Lischer, 2010). Mean expected heterozygosity for each sample as a response variable depending on location‐specific environmental factors (summer salinity, winter salinity, summer temperature, winter temperature, MPA status, and distance from the North Sea following the coastline (see Table 1)) were modeled using multiple regression in R, after which the significance of each explanatory variable was tested using ANOVA. To do this, environmental data for the top 6 m were extracted from the Rossby Centre Oceanographic circulation model (Meier, Döscher, & Faxén, 2003) and averaged across 1995–2004 for all locations except for W‐1, which was outside the domain of the model. For the location W‐1, empirical data from the ICES database (ices.dk) for depths ≤ 5 m were averaged for the years 1995–2004 for the nearest locations present in the database. Summer and winter values were averages for June–August and December to February, respectively. Summer and winter salinity were found to be strongly correlated, and thus, only summer salinity was used as a factor. For all other factors, variance inflation factors were < 3, and thus, they were kept in the model.

### 2.4 Principal components analysis

An identity‐by‐state (IBS) distance (1—IBS) matrix was calculated on an individual basis from the full 33,774 SNP dataset, using plink 1.9 (Purcell et al., 2007). A Canonical Analysis of Principal coordinates (CAP) was performed using the Vegan R package, assigning multidimensional coordinates to each isopod individual. This method is similar to a PCA or an MDS, but it rotates the eigenvector axes in such a way as to maximize the among‐sample differences. Also, a test of the significance of sample as a factor was tested through an ANCOVA within the Adonis R package.

To identify loci potentially affected by natural selection, a global *F*_ST_ outlier analysis was performed using two methods, Bayescan v 2.1 (Foll & Gaggiotti, 2008), using a cutoff value of an FDR‐corrected p‐value of 10^–4^ to determine significance, and OutFLANK (Whitlock & Lotterhos, 2015), which is more stringent than BayeScan in outlier calling, and thus minimizes false positives. The output from the two methods was then examined for overlap. IBS matrices and CAP analyses were calculated separately for the shared outliers and nonoutliers, in order to determine whether outliers had disproportionate or distortive effects on the genetic structure analyses. As outliers and nonoutliers showed similar patterns, outliers were kept in downstream analyses.

### 2.5 Geographically explicit population inference

Genotype likelihoods for all SNP sites were extracted from the vcf files using bcftools, which were input to NGSadmix (Skotte, Sand Korneliussen, & Albrechtsen, 2013) for population cluster analysis. NGSadmix uses a probabilistic framework to infer ancestry. *K* of 2–32 genetic clusters were evaluated, with 10 replicates of each. The output q‐matrices of NGSadmix were combined for each *K* and plotted as barplots using CLUMPAK online (http://clumpak.tau.ac.il/index.html). Cross‐validation scores were examined for each *K* to infer the most probable number of population clusters.

The software “conStruct” (Bradburd, Coop, & Ralph, 2018) was used to generate pie charts of admixture proportions at *K* = 3 (given by the most significant drop in NGSadmix cross‐validation scores, see results). conStruct uses a Bayesian MCMC algorithm to estimate the posterior distribution of admixture proportions. The input for conStruct was sample allele frequency for each SNP, and the MCMC analysis was run with two chains, 50 000 iterations, in the nonspatial mode of the software. In order to extrapolate NGSadmix‐estimated ancestry coefficients (proportion of the genome belonging to the different clusters) on geographic scales, the R package TESS (Caye, Deist, Martins, Michel, & François, 2016) was used. We chose *K* = 12 genetic clusters for plotting (given by a plateau in NGSadmix cross‐validation scores, see Results). A map of the Baltic Sea was downloaded from https://maps.ngdc.noaa.gov/viewers/wcs-client/, from which a grid was generated with a constraint on elevation from 0 m to −30 m (grid size 1,852 × 1,852 m). The constraints were chosen to account for variability in depth within each grid cell, as well as for plotting reasons. Ancestry coefficients for the 12 different clusters were then geographically imputed and plotted on the map in different color scales.

Finally, variability in genetic diversity was modeled and extrapolated geographically using the software package EEMS (Petkova, Novembre, & Stephens, 2015). This software uses an isolation‐by‐distance model with stepping stones (“demes”) as a null model in order to infer geographic areas of high and low genetic diversity using the parameter *q*, a parameter which reflects the expected genetic differences among two individuals collected at the same site (Petkova et al., 2015) (not to confuse with the ancestry coefficients from the NGSadmix analysis above, which are also denoted “*q*”). A full‐rank distance matrix was generated by imputing missing genotype values with observed mean genotype at each SNP site, as implemented by the “bed2diffs_v2” tool distributed with the EEMS software. Geographic coordinates from 86 vertices specifying a polygon enclosing the Baltic Sea were extracted from Google Earth (earth.google.com/web/), after which the EEMS software was run in three separate mcmc chains, each with default lengths (2 M iterations, burn‐in 1 M iterations, writing every 10 K iterations to file), using 300 demes. The output was examined for convergence and plotted on a map of Europe using the rEEMSplots R package (distributed with EEMS).

### 2.6 Biophysical model

A biophysical model was used to estimate dispersal probabilities and multigenerational connectivity calculated from stepping‐stone dispersal across generations. All results below from the biophysical model are referred to as “connectivity,” to be distinguished from results obtained from genetic data, which are referred to using the terms “gene flow” and “population structure.” The biophysical model is based on the oceanographic circulation model NEMO‐Nordic that produces water velocity fields with spatial resolution of 3.7 km in the horizontal and 3–12 m in the vertical, and a temporal resolution of 3 hr (for details, see Hordoir et al., 2019). The velocity fields are used by the Lagrangian particle tracking model TRACMASS (de Vries & Döös, 2001) to estimate dispersal from a location *i* to a location *j* where results are conveniently summarized as a normalized connectivity matrix with elements specifying the dispersal probability between all locations in the domain (Jonsson, Nilsson Jacobi, & Moksnes, 2016). The particle tracking model calculated the dispersal from and to 34,036 locations where particles were parameterized to mimic dispersal of *I. balthica* assuming the following traits and conditions: Reproduction occurs between April and September, drift or swimming of adults or juveniles is in the surface water (0–2 m), and the total duration of the dispersive phase was here approximated to be ca. 5 days. Dispersal of *Idotea* spp. is not well known but is believed to be short (Leidenberger et al., 2012), with rare long‐distance rafting events, motivating the choice of dispersal time. The trait combination assumed here possibly leads to an overestimation of realized dispersal and should be seen as a maximum rate. We also only considered model grid cells with a mean depth less than 30 m, as in the genetic analyses above. In total, the dispersal simulations of *I. balthica* included 34 million particles.

To identify potential dispersal barriers, we applied a clustering method (Nilsson Jacobi, André, Döös, & Jonsson, 2012) based on the connectivity matrix. Identification of geographic areas separated by partial dispersal barriers is here formulated as a minimization problem with a tuneable penalty term for merging clusters, which makes it possible to subdivide areas with varying degrees of dispersal restrictions. Areas that have an internal connectivity above the dispersal restriction are color‐coded, and the transitions of colors thus indicate partial dispersal barriers. From the dispersal matrix, we produced three maps of subdivided connectivity clusters separated by partial dispersal barriers with mean probabilities of crossing barriers of 0.001, 0.002, and 0.003, producing 7, 13, and 24 connectivity clusters, respectively.

Multigenerational connectivity was calculated from the dispersal connectivity matrix by multiplying the matrix with itself for each generation (Jahnke et al., 2018). This procedure calculates the stepping‐stone dispersal when dispersal probability for all possible routes is summed across all generations for 64 generations. With a mean dispersal distance of approximately 25 km per generation, simulations for 64 generations should ensure potential stepping‐stone connectivity and gene flow on the scale of the Baltic Sea in the absence of barriers.

### 2.7 Genetic versus. biophysical connectivity

The correlation between genetic differentiation (pairwise *F*_ST_) and multigenerational connectivity estimated from the biophysical model was tested with Mantel tests (Mantel, 1967). Correlations with both untransformed and logarithmically transformed (log_10_(*x* + 1e‐50)) connectivity matrices (64 generations) were tested. Because pairwise *F*_ST_ data are symmetric, we extracted symmetric connectivity matrices by using either the minimum connectivity (minimum of *i* to *j* and *j* to *i*), the maximum connectivity, or the mean connectivity. In addition, distributions of pairwise *F*_ST_ values within each region and between regions were plotted as boxplots in R, and a regression analysis was performed to examine potential isolation‐by‐distance patterns between mean connectivity and pairwise *F*_ST_.

## 3 RESULTS

### 3.1 Accuracy of genotyping

The 2b‐RAD libraries generated *µ* = 8.64 M reads ± *SD* 3.72 Mreads per individual, and mapping rates (not including multiple hits) were *µ* = 20.23% ± *SD* 2.26% (Table S1). This rather low percentage is not unexpected given the short nature of the 2b‐RAD tags and the incomplete genome assembly used (NG50 = 12.9 kb based on assumed haploid genome length 1 Gb; 338 k contigs; 1.54 Gb total length; 80% BUSCO completeness using Metazoa reference). Using 25,329 SNPs that had identical genotypes across all replicate pairs of individuals as a “true” training set for the VQSR, we estimated the true transition/transversion ratio (*T_i_*/*T_v_*) to be 1.348. By examining different truth‐sensitivity tranches, we could observe a sharp drop in *T_i_*/*T_v_*at above 95% truth sensitivity, so we used 95% as the cutoff tranche for SNP filtering. After filtering for highly heterozygous (likely lumped paralogous) loci, and thinning the dataset to one SNP/ RAD fragment, we were left with 57,641 SNPs, and after filtering out poorly genotyped sites, the final genotype dataset consisted of 33,774 SNP sites, genotyped at a minimum of 80% of 575 individuals (Table S1) from the 31 different samples (Figure 1; Table 1). Genotyping correspondences among technical replicate individuals were high, *µ* = 97.70% ± *SD* = 2.49% (Table S2), and missing data were evenly distributed across samples, with only 9 loci not genotyped in any individual in one sample, and no loci with all missing data from more than one sample (Table S3).

### 3.2 Among‐sample summary statistics and differentiation

Overall, considering all loci, *F*_IS_ values were close to zero in all 31 samples, indicating within‐location panmixia (mean = 0.020 ± *SD* 0.029; *n* = 31; Table S4).

Pairwise *F*_ST_ values among samples ranged from 0.001 to 0.068 (mean = 0.025 ± *SD* 0.012; *n* = 465) (Table S5 above diagonal). In general, *F*_ST_ values among samples within a region were smaller than *F*_ST_ values among samples from different regions, with the highest values between SBO samples and W/GFE samples (mean = 0.053 ± *SD* 0.006; *n* = 32) Figure S1). In an MDS plot (Figure S2), axis 1 and axis 2 together explain much of the variance in pairwise *F*_ST_ (61% and 24%, respectively). In this analysis, the Swedish Bothnian Sea samples (SBO‐1 and SBO‐2) in particular diverge strongly from all others along both primary axes. There was significant genetic differentiation (based on sample allele frequencies) among most samples (Fisher's exact test *p* « .001; Table S5 below diagonal). Exceptions to this pattern were found in the Skagerrak/Kattegat/Danish straits area (W‐1‐9; marked blue in Table S5) and along the northern part of the Finnish Bothnian Sea coast (FBO‐1‐6; marked green in Table S5), where many samples were not significantly differentiated from each other, indicating that these samples were taken from the same population. Surprisingly, the Estonian samples collected from three of the sites (GFE‐4 to GFE‐6, and in particular, GFE‐5) were genetically similar both to eastern Finnish sites (FBO‐5,6) and to sites from Kattegat/Skagerrak/Belt Sea (W‐1–8) and western Baltic Proper (SBA‐2,3) samples. Unfortunately, the fragmented reference genome used did not allow for a closer examination of *F*_ST_ patterns along chromosomes.

**Figure 2:**
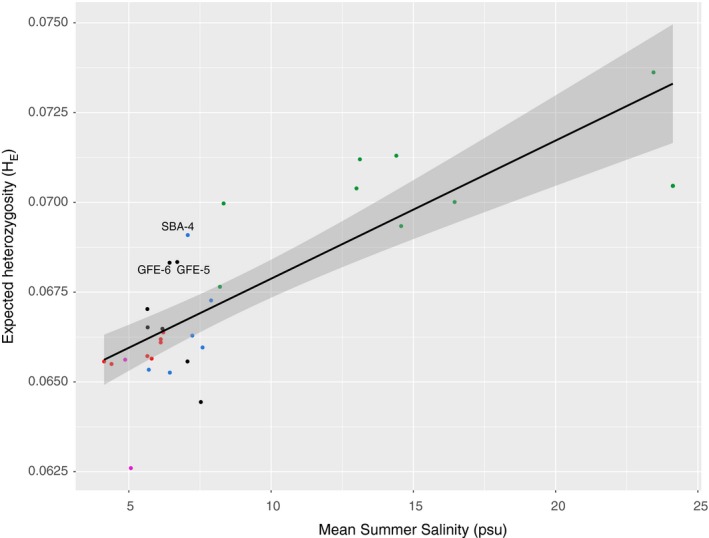
Expected heterozygosity (*H*_E_) as a function of salinity in the Baltic Sea (*p* = 8.5E‐08), with dots colored by region

Mean expected heterozygosity per sample (Table S4) was strongly correlated with the location's salinity (Figure 2, ANOVA *p* = 5.36E‐08), primarily due to a major drop at the North Sea–Baltic Sea transition zone (without W samples, *p* = .10). However, there was no significant effect of mean summer (*p* = .28) or winter (*p* = .09) temperature, or with geographic coastline distance from the North Sea (*p* = .56). Heterozygosity was also not significantly different between HELCOM MPA and non‐MPA locations (*p* = .26) (Table S6).

### 3.3 Individual‐based inference of genetic admixture and population structure

Our canonical analysis of principal coordinates (CAP) was based on an IBS distance matrix calculated for all individuals in all samples. BayeScan identified 487 *F*_ST_ outliers across all samples (Figure S3a), and OutFLANK identified 170 *F*_ST_ outliers, where 111 of the outliers overlapped across both methods. Separate CAP analyses for outliers and nonoutliers (Figure S3b‐d) revealed similar clustering patterns, although there was less separation along the two principal axes in both outlier datasets, which could be due to the lower number of SNPs used, or to the structuring of the outlier loci. Exceptions from the overall similarity were the two Swedish Bothnian Sea samples (SBO‐1 and SBO‐2), being much less deviant from the rest of the samples when using outliers only than when the analysis was based on nonoutliers. Mean summer salinity was an equally strongly significant factor in the CAP axis 1 loading in both outliers and in nonoutliers (*p* < 2E‐16; Table S6). Due to the similar patterns observed in both types of loci, all loci were analyzed together for inferring genetic structure.

**Figure 3:**
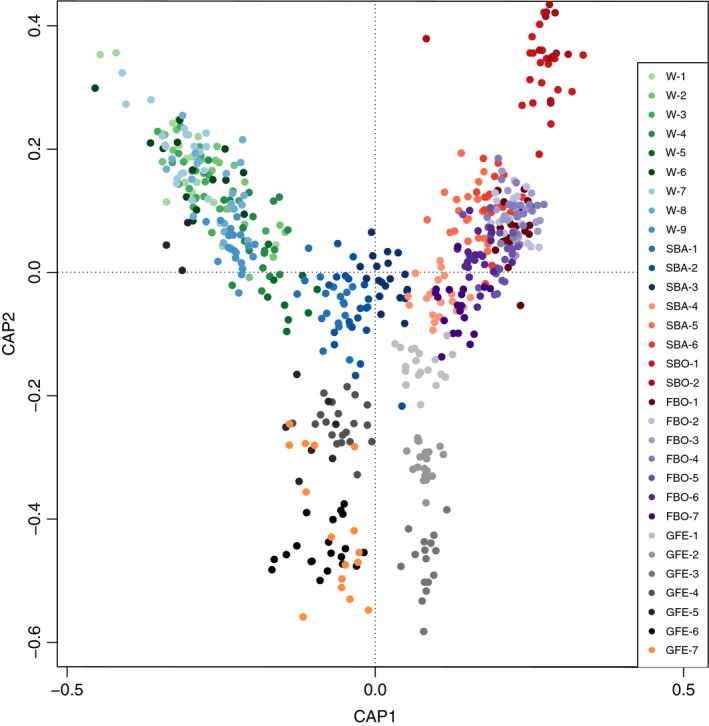
Constrained ordination plot of identity‐by‐state distances among all *I. balthica* individuals used in this study

The combined CAP analysis clearly separated samples, or groups of samples, from each other, matching the geographic distribution of the collecting locations almost perfectly (Figure 3). Indeed, sample was a strongly significant factor, explaining 12% of the genetic variance (ANCOVA, *p* = .001; Table S6). Just as in the sample‐level analysis of allele frequency differentiation above, samples collected from different regions were always separated from each other, while in some cases standard deviations from samples collected in the same region overlapped in the CAP. Individuals in samples from the Skagerrak/Kattegat/Danish straits area (W‐1,2,3,6,7,8) completely overlapped each other in the CAP, as did individuals from the northernmost Finnish Bothnian Sea samples (FBO‐1,2,3,4), again indicating panmictic populations in these two regions. Along the coast of the Swedish Baltic Sea Proper (SBA) and the southern part of the Finnish Bothnian Sea (FBO‐5,6,7), there was a step‐wise divergence (see differentiation along CAP axis 1, Figure 3), while the Gulf of Finland and Estonian samples diverged along CAP axis 2, as did the Swedish Bothnian Sea populations (although in the other direction of the axis), indicating that each of these samples were collected from a distinct population. Thus, while most samples were distinct in the CAP, there was also an overall pattern of divergence along three main directions in CAP1 and 2; one in the west (with negative CAP1 and positive CAP2 loadings), one in the north Baltic/Bothnian seas (with positive loadings on both CAP1 and 2), and a third one in the Gulf of Finland/Estonia (negative CAP2 loadings). In the Gulf of Finland, samples GFE‐1,2,3 were separated from Estonian samples GFE‐4,5,6,7 along CAP axis 1. Interestingly, sample GFE‐5 (Sarve, Estonia) also showed a very large spread among individuals in both principal axes of the CAP, with some individuals being very close to individuals from the Western Baltic, while others were more similar to individuals found in other Estonian samples.

**Figure 4:**
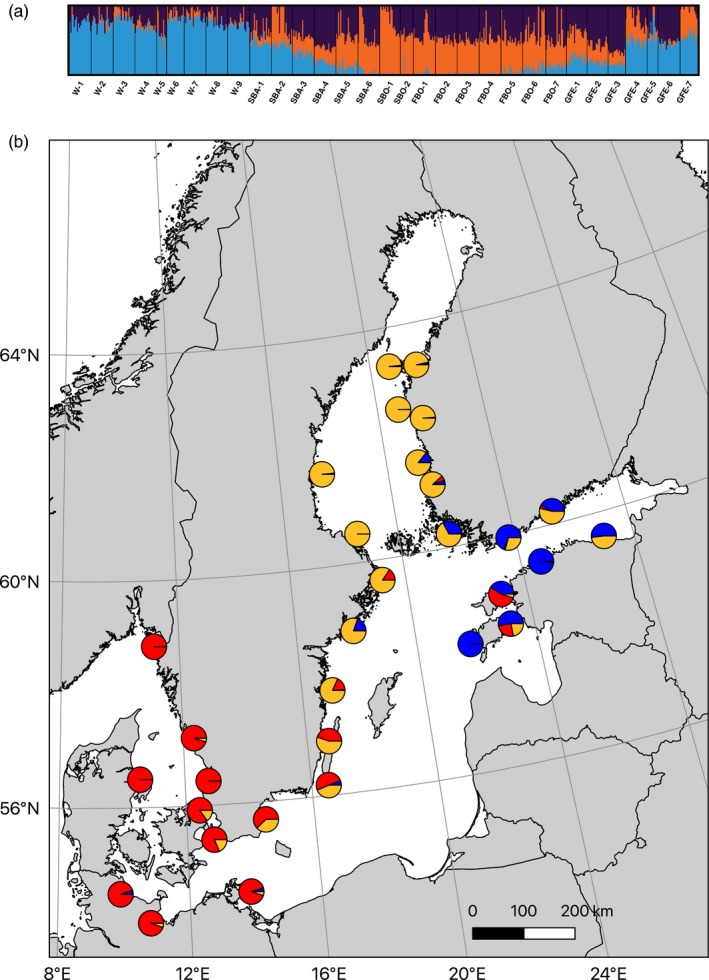
(a) Admixture plot at *K* = 3. (b) Pie charts of admixture coefficients inferred by conStruct (*K* = 3), plotted on a map of the Baltic Sea

The admixture analysis was run for *K* = 2–32 (Figure S4), and examining the cross‐validation scores (Figure S5), the most significant drop was found from *K* = 2 to *K* = 3, and a plateau was found at *K* = 11–12 (after an increase in the cross‐validation value at *K* = 10), although the software did identify more fine‐scale population structure also at higher *K* values. Thus, we chose to focus on *K* = 3 and 12. At *K* = 3, western samples, North Baltic/Bothnian Sea ones, and Estonian ones (excluding the Estonian GFE‐3 sample) formed three main clusters (Figure 4a). In admixture pie charts generated by conStruct (Figure 4b), the three main clusters were clearly associated with the different regions, with the red cluster being dominant in the west, the yellow dominant in the Baltic Sea, and the blue cluster dominant in the Gulf of Finland. Interestingly, the red cluster was also observed in samples GFE‐5 and GFE‐6, in Estonia, while the Swedish southeast coast consisted of a gradual shift of genetic material from the red to the yellow cluster, which could be an indication of a possible secondary contact zone among historically separated lineages.

**Figure 5:**
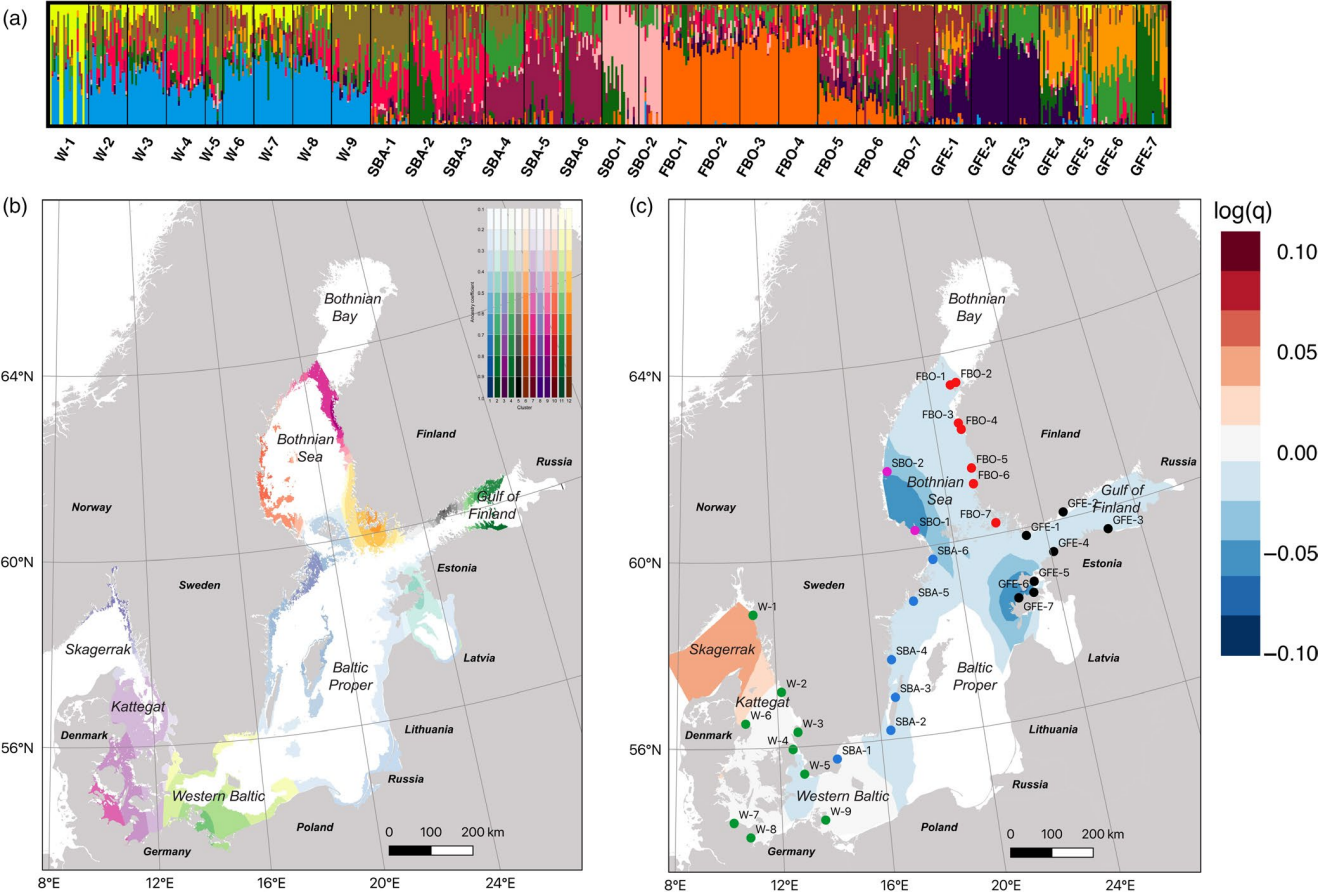
(a) Admixture plot at *K* = 12. (b, c) Spatially inferred genetic structure. (b) Admixture coefficients interpolated geographically, with population clusters in different color scales (*K* = 12). Admixture coefficients are illustrated in different color scales, approaching 1 in dark shades, and lower scores in progressively lighter ones. Regions with steep color gradients can be thought of as areas with dispersal barriers, separating genetically divergent populations; (c) within‐deme effective genetic diversity q (on the log10 scale), a parameter that reflects the expected genetic differences among two individuals collected at the same site, extrapolated geographically, with higher than mean diversity in red and lower in blue

At *K* = 12, a pattern of step‐wise divergence of samples could be seen along the Swedish west and south coast (Figure 5a). Western Estonian samples clearly separated out from all others. In the west, W‐4 and W‐9 clustered together, W‐1 was separated, while all other samples grouped together. In the east, there was one large cluster all along the Northern Bothnian Sea coast of Finland, one cluster in the Gulf of Finland, and two on the Swedish coast: SBA‐5,6 and SBA‐4, respectively, while the geographically intermediate samples FBO‐5 and FBO‐6 appeared to contain a mix of genetic material from the Swedish and the Finnish clusters. By geographically extrapolating admixture coefficients at K = 12 using the TESS algorithm, the geographic distributions of the different genetic clusters, and the main boundaries between them, were plotted in different color scales on a map of the Baltic Sea (Figure 5b). As there were no samples collected in Poland, Lithuania, and Latvia, nor on the island of Gotland, the imputations in these areas are subject to caution. Nevertheless, the large‐scale structure seen in the map indicated at minimum 10 geographically separated populations in the study area, located in: 1. Skagerrak; 2. Kattegatt (including the Danish straits); 3. The Western Baltic (Sweden/Germany), 4. The Swedish Baltic Proper; 5. The Swedish Bothnian Sea; 6. Southwestern Finland (including the Archipelago Sea); 7. The northern Finnish Bothnian Sea coast; 8. The Helsinki area; 9. The deep Gulf of Finland; 10. The Estonian islands.

Finally, within‐deme effective genetic diversity (*q*) (Petkova et al., 2015) was higher in the Skagerrak area than in the Baltic Sea, with particularly low values on the Swedish Bothnian Sea coast (SBO‐1 and SBO‐2) and in western Estonia (GFE‐7) (Figure 5c).

### 3.4 Biophysical model predictions of connectivity

**Figure 6:**
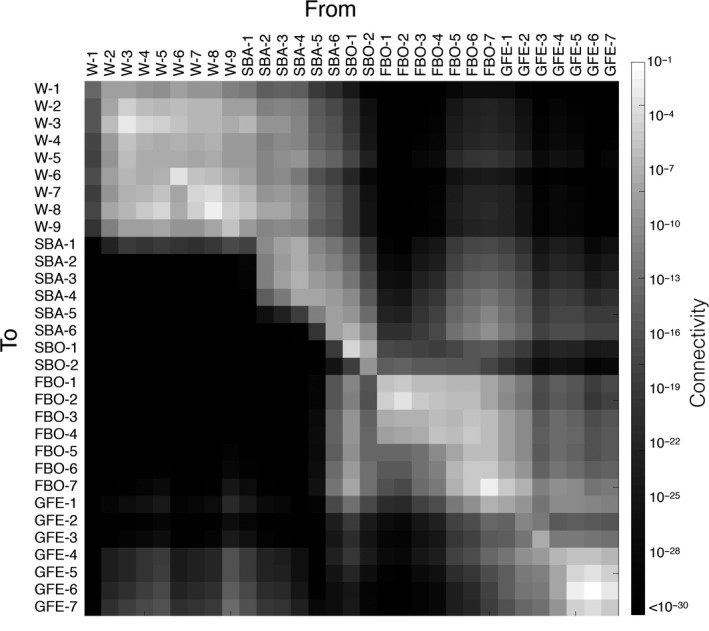
Biophysical connectivity matrix among the sites used in this study, based on a multigenerational iteration of the particle tracking model

The asymmetrical multigeneration connectivity matrix (64 generations) based on the biophysical particle model (Figure 6, Table S7) showed that there were certain regions with high internal connectivity, which were less well‐connected to each other. The western Baltic, the Finnish Bothnian Sea, southern Finland, and western Estonia were four such regions. In contrast, along the Swedish southeast coast, isolation‐by‐distance was expected from the model. Connectivity was in general symmetrical, with a few interesting exceptions: Connectivity was several orders of magnitude higher from the Finnish coast (FBO‐5,6,7) to the Swedish East coast (SBA‐5,6) than in the other direction. Instead, there was stronger connectivity from the northernmost locality in the Swedish Bothnian Sea (SBO‐1) over to the Finnish Bothnian Sea sites. Along the Finnish Bothnian Sea coast, connectivity was also asymmetrical according to the model, with higher values going northward than southward, and along the Swedish south coast, there is more transport from the Baltic toward the Kattegat/Skagerrak region than in the other direction. Interestingly, the particle model also indicated a high connectivity from the western Baltic to western Estonia, via Poland, Lithuania, and Latvia, resulting in a significant amount of connectivity from the Swedish south coast (W‐5, SBA‐1) to the western Estonian sites.

**Figure 7:**
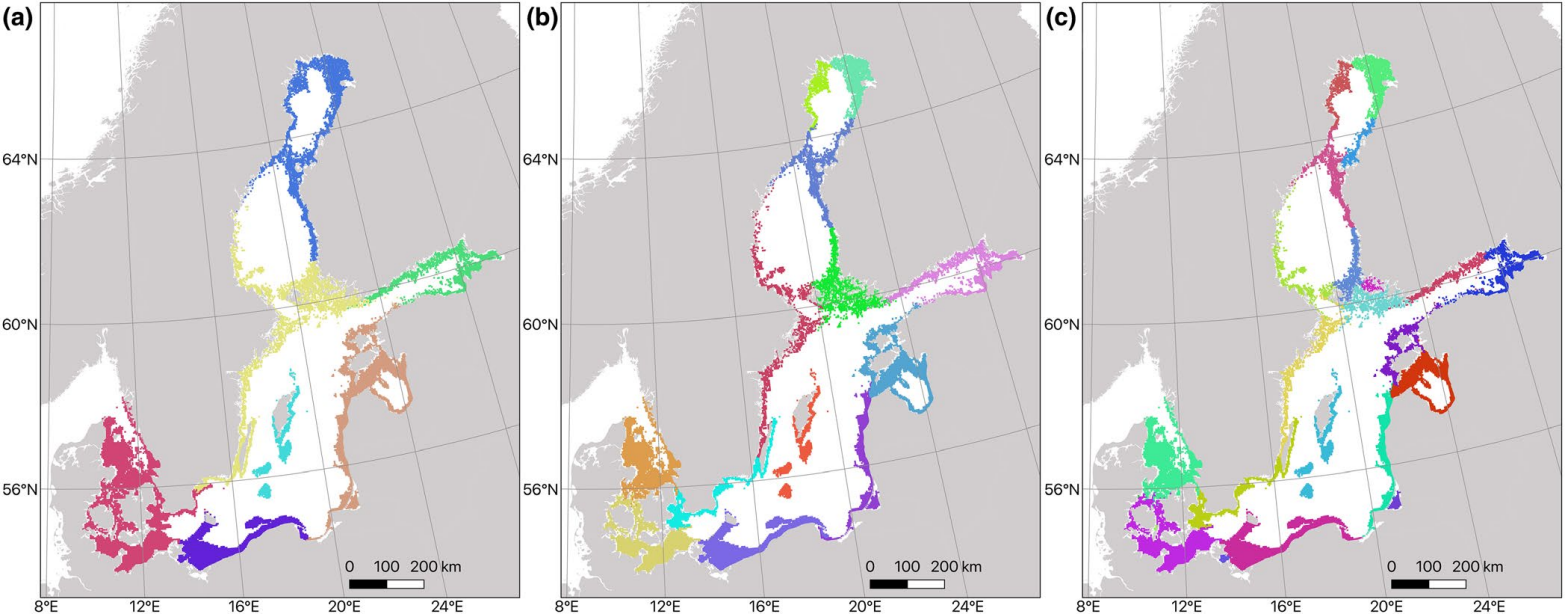
Connectivity barrier inference based on the biophysical model of dispersal, clustering areas of high connectivity marked as different colors. Threshold for barrier identification ranges from low dispersal probability between regions (0.001) in panel (a) intermediate (0.002) in panel (b), and high (0.003) in panel (c)

The barrier analysis, which is a way to project the multigeneration connectivity matrix in a geographic dimension, also identified regions with high internal connectivity within the Baltic Sea separated by barriers with high resistance to dispersal (Figure 7). The number of regions varied according to the selected threshold of allowed mean dispersal among regions. The model with the highest threshold (Figure 7c) most closely matched our genetic data, in that it identified the separation between the Swedish Bothnian Sea samples and other (including nearby) samples. However, this cutoff value also separated Finnish Gulf of Finland samples from Estonian ones, which was not clearly evident in the genetic data (although the yellow conStruct cluster is largely restricted to the Gulf of Finland); here, the barriers in Figure 7b better matched the genetic data. The barrier analysis averages connectivity values to and from sites and thus inaccurately represents instances of highly asymmetric gene flow, which could explain this discrepancy.

**Figure 8:**
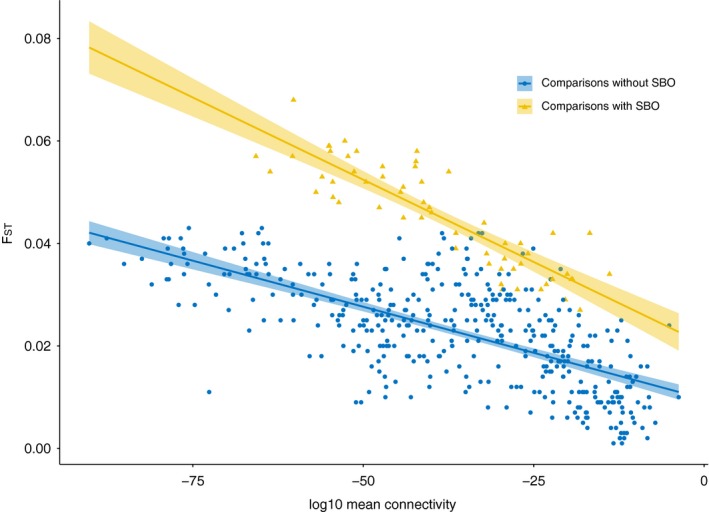
Pairwise *F*_ST_ as a function of log10 mean connectivity. Two separate correlations are found for pairwise comparisons involving Swedish Bothnian Sea (SBO) samples (yellow triangles; *p* < 2E‐16; *R* ^2^ = 0.76) and pairwise comparisons among all other samples (blue dots; *p* < 2E‐16; *R* ^2^ = 0.44)

Overall, the biophysical model (mean connectivity) was strongly correlated with pairwise genetic distance (*F*_ST_) (Mantel *p* < 1E‐5, *R* = −0.45). However, the regression analysis identified two separate isolation‐by‐distance (IBD) correlations in the dataset: one involving pairwise comparisons including one of the two SBO samples (*R* ^2^ = 0.76) and one with all remaining pairwise comparisons (*R* ^2^ = 0.44) (Figure 8; Table S6).

## 4 DISCUSSION

### 4.1 Concordance between biophysical connectivity and genetic structure

The population genomic data produced here shows the presence of genetically differentiated populations of *Idotea balthica* within the Baltic Sea. In some of the regions, there are significant population differences on scales of less than 100 km. This contrasts to patterns in some fish species such as sticklebacks (DeFaveri, Jonsson, & Merilä, 2013) and herring (Lamichhaney et al., 2012) where populations are genetically structured at broad geographic scales in the Baltic Sea, while in other fish species, such as perch and whitefish, genetic differences are found also on relatively small scales (Olsson, Florin, Mo, Aho, & Ryman, 2012; Olsson et al., 2011). Instead, the strong genetic structure in *I. balthica* is similar to the pattern present in the seaweed *Fucus vesiculosus* (Ardehed et al., 2016), which has an overlapping distribution with *I. balthica* and provides food and shelter to the isopod (Kotta et al., 2019). Further, the genetic patterns match the biophysical connectivity model closely, suggesting that passive dispersal is an important factor influencing the genetic structure in this species. Without pelagic larval dispersal, long‐distance dispersal is most likely through rafting of adult or juvenile individuals associated to *Fucus* or possibly other seaweeds (Clarkin, Maggs, Arnott, Briggs, & Houghton, 2012; Thiel & Gutow, 2005; Winston, 2012). Although we observed a few individuals with partly western ancestry at the Estonian GFE‐5 site, which perhaps support the hypothesis of long‐distance dispersal along the southern Baltic Sea coast, our data suggest that long‐range dispersal of *I. balthica* in the Baltic Sea is rare. One potential reason could be the high predation risk—*Idotea* spp. are usually found in high numbers only in thick algal belts, where they are protected from predation from multiple fish species (Merilaita, 2001). So, any long‐distance swimming or rafting with parts of algal thalli would entail a high risk of being eaten. The biophysical model of connectivity included pelagic drift during 5 days. Although this may overestimate the dispersal capability of *I. balthica*, the model still suggests several areas acting as physical barriers to dispersal as visualized in the barrier analysis (Figure 7). The barrier analysis with a step‐wise increase of the allowed dispersal probability (0.001–0.003) across barriers indicates the strength of barriers and the robustness of connectivity clusters (Nilsson Jacobi et al., 2012). Some dispersal barriers are consistent across a range of dispersal thresholds (e.g., between western Estonia and the Gulf of Finland), whereas other barriers are more labile (e.g., along the Swedish east coast). In general, the barrier analysis alone predicted the population genetic structure showed by the genetic data well (Mantel test r = −0.45). However, we cannot rule out contributions from demographic history (Gagnaire et al., 2015; Le Moan, Gaggiotti, et al., 2019b), local adaptation (Johannesson et al., 2011), and/or genetic incompatibilities (Bierne et al., 2011), in shaping the population genetic structure of the species. In particular, at K = 3, there is a pattern of three main clusters, which could be a signal of anciently separated lineages, one in the west, one in the north, and one in the east. This pattern can also be observed as separations along three different directions in the CAP analysis, with central Baltic samples being located in the middle being a sign of potential introgression among the clusters. However, the isolation‐by‐distance analysis does not seem to indicate larger genetic distances between‐cluster variation than within‐cluster variation in *F*_ST_, indicating that more sensitive methods (e.g., site‐frequency spectrum based analyses) need to be used to examine patterns arising from historical demographic events.

The Swedish Bothnian sea samples (SBO‐1,2) show low genetic diversity and are genetically isolated from all other samples with high pairwise *F*_ST_ estimates. They are also found to be significantly more distant from other samples in the isolation‐by‐distance regression analysis. This is partly at odds with the barrier analysis, which indicates that dispersal along the Swedish coast include these localities, except in the most stringent setting of the barrier analysis (Figure 7c). However, when the possibility for asymmetric dispersal is also considered (heatmap in Figure 6), the biophysical model shows that SBO‐1 and SBO‐2 are almost isolated from incoming migrants, although they might act as sources to locations mainly along the western coast of Finland. It seems that the patterns of the oceanic currents north of the Åland archipelago provide an effective barrier to westward dispersal in this area, instead directing transfer of individuals from Sweden to the Finnish Archipelago sea, and then northward along the Finnish Bothnian Sea coast. Another inconsistency is that the biophysical model suggests dispersal from Finland to Sweden in the transition area between the Bothnian Sea and the Bothnian Bay (the “North Quark”; see Figure 1). *Idotea balthica* is quite common on the Finnish side (FBO‐1,2), and this area is shallow and may provide good stepping‐stone dispersal for shallow‐water organisms, so even species with limited dispersal ability should be able to cross over to Sweden from the Finnish side. However, isopods are not found on the Swedish side (Leidenberger et al., 2012). In fact, our SBO‐2 site, approximately 300 km further south, is the most northern point of the species distribution on the Swedish coast. There are available habitats (*Fucus* belts) north of this site, although the distribution of *Fucus* spp*.* becomes increasingly patchy. Niche modeling studies (Kotta et al., 2019; Leidenberger, Giovanni, Kulawik, Williams, & Bourlat, 2015) indicate that the northern Bothnian Sea coast should be inhabitable to the isopods, rendering the reason for their absence in this area an interesting topic for future research.

### 4.2 Main population units of *Idotea balthica* in the Baltic Sea

*Idotea balthica* along the Finnish Bothnian Sea coast is one large continuous population in the northern part (FBO‐1,2,3,4). As prevailing currents are northward bound along the Finnish coast, it is likely that gene flow is mainly in the south–north direction (from FBO‐5,6). Possibly, the northernmost Finnish population might be a sink population with low reproductive output and few dispersive juveniles, which would add to the explanation of lack of dispersal to the Swedish side. The strong genetic separation of the Swedish and Finnish Bothnian Sea coasts can be compared to the genetic patterns observed in *Fucus*. While *F. radicans* show weak structure throughout the region due to dominance of a few large clones, *F. vesiculosus* is differentiated both between Sweden and Finland and also among sampling sites within both countries (Pereyra et al., 2013). Again, it seems as if the herbivore and its host species show similar structuring patterns, and an interesting future avenue for research would be to compare current gene flow and colonization history of both species more closely.

The Estonian coast is divided into two distinct regions: the Gulf of Finland area, which is genetically more similar to the Finnish side of the Gulf of Finland, and western Estonia, represented by the islands of Hiiumaa and Saaremaa. Interestingly, both the pairwise *F*_ST_ MDS plot (Figure S2) and the CAP analysis (Figure 3) indicated gene flow from southern Sweden and eastern Germany to Estonia. The biophysical model also indicated a relatively high potential for dispersal in this direction (Figure 6). However, the Baltic Sea coastline of Poland, Latvia, and Lithuania is sandy and lacks *Fucus*, which would provide an effective barrier to gene flow for *Idotea* unless the isopod can use other habitats (e.g., *Zostera* beds) as stepping stones for dispersal. The Polish, Latvian, and Lithuanian coasts have historically supported extensive seagrass beds, although almost all of those are gone today (Boström, Baden, & Krause‐Jensen, 2003). Further sampling of *Idotea* along these shores will be necessary in order to illuminate the patterns of gene flow here.

Along the Swedish coast, starting from Öresund (W‐4), samples show a strong step‐wise isolation‐by‐distance divergence going in to the Baltic. Such a pattern is expected following a colonization front moving into the Baltic Sea along the coast, with founder effects leading to increasing levels of differentiation (Excoffier, Foll, & Petit, 2009). Alternatively, this pattern could be explained by a historical separation into three separate lineages in the Baltic Sea, with secondary contact generating a gradual introgression pattern along the Swedish coast (Bierne et al., 2011). The CAP and construct analyses seem to support this interpretation, although the isolation‐by‐distance regression only singles out the Swedish Bothnian Sea samples as more divergent than predicted by connectivity only. A third possibility is that divergent selection on either side the steep salinity gradient of the transition zone cause the observed differentiation. Modeling the demographic history of the species and the timing of the range expansion into the Baltic Sea along with reciprocal transplant experiments (to test for local adaptation to different salinities) would be beneficial in order to gain a deeper understanding of the contribution of the individual factors during establishment of the observed genetic structure.

### 4.3 Reduced genetic diversity

The low heterozygosity in *I. balthica* in the Baltic Proper corresponds to a 12% drop compared to the most diverse sample on the Swedish west coast (W‐1). Interestingly, this corresponds very well to the average drop in nuclear genetic diversity of Baltic Sea populations of various marine species (11%–12%) (Johannesson & André, 2006). One possible explanation is the influence of genetic drift due to periodically low population sizes purging diversity in the Baltic Sea (Johannesson & André, 2006). However, some observed patterns could also be explained by secondary contact and introgression among historically separated lineages enhancing diversity locally. Secondary contact zones in this area have been recently described for three species of fish (Le Moan, Gaggiotti, et al., 2019b). However, as the genomic reference used in this study was highly fragmented, we could not investigate genomic patterns of introgression in our dataset. Hopefully, future improvements in genome sequencing will allow for such studies in the future.

Within the Baltic Sea, the western part of Estonia stands out as a region of lower genetic diversity, in large part due to low diversity in the sample GFE‐7 (Figure 5c; Table S4). GFE‐7 was collected on the highly exposed west coast of Saaremaa island, where also the biophysical connectivity analysis indicates a lower input of propagules compared to other sites along the Estonian coast (Figure 6). Along the isolated Swedish Bothnian Sea coast, diversity is also low, especially in sample SBO‐1 (Gräsö island). These locations could be considered important from a MPA network perspective, as local reductions in diversity are due to either low population sizes, low migration, or a combination of the two factors.

### 4.4 Management and conservation implications

The reduced within‐species genetic diversity, and the need for adaptations to the rapidly changing Baltic Sea, call for management and conservation actions (Johannesson et al., 2011). Marine protected areas (MPAs) would be one way to conserve genetically diverse and hence valuable populations. Despite the fact that the Baltic Sea hosts a number of MPAs (each with unique historical and regulatory conditions), we found no significant difference in genetic diversity in populations of *I. balthica* within and outside of HELCOM marine protected areas. This lack of relationship between MPAs and within‐species genetic diversity has been documented also in other species in the Baltic Sea (Wennerström et al., 2017), although there are documented positive relationships in other areas of the world (Lester et al., 2009). According to both the United Nations Convention of Biological Diversity (CBD 1993) and the European Union Marine Strategy Framework Directive (Directive 2008/56/EC), designation of protected areas should be planned with regard to conservation of biodiversity, including within‐species genetic diversity (Laikre et al., 2016). On the Baltic Sea scale, the Helsinki Commission also recognizes within‐species diversity as an important factor in determining ecosystem resilience (HELCOM, 2009). However, this objective has to date not been implemented in management plans and in the design and location of the present MPAs in the Baltic Sea (Laikre et al., 2016). Useful information of species genetic diversity is currently accumulating, but managers hesitate to use genetic diversity in day‐to‐day operations as they find this type of information difficult to interpret (Sandström, Lundmark, Andersson, Johannesson, & Laikre, 2019). One way to overcome this problem may be to provide easy‐to‐interpret maps of genetic diversity and population subdivision, which can be used to implement management efforts in several ways: First, the genetic clusters identified in Figure 5b can be considered as a good starting point for defining management units in this organism, where the Kattegat/Skagerrak/Danish Straits area, and also the west coast of Finland might be managed as single units, while in the other parts of the study area the observed population divergence indicates a need for more local management units. It is important to note, however, that this study does not include data from the island of Gotland, nor from the coasts of Poland, Latvia, and Lithuania. In these areas, there are (rare) historical records of *I. balthica* (Leidenberger et al., 2012). Large parts of the southern Baltic coast consist of sandy areas with limited *Fucus*‐habitat, yet *I. balthica* can also be found in eelgrass (*Zostera marina*) beds, so it cannot be excluded that there are additional genetic clusters present there. Further sampling efforts will be necessary in order to resolve this issue.

Second, highly diverse populations of high priority for conservation can be identified from the genetic data. Genetic diversity is a proxy for the long‐term adaptability of a population and can also be considered a sign of a relatively large and stable population size. In this dataset, sites with higher than average genetic diversity (as measured by expected heterozygosity) while considering the overall salinity effect can be found on the eastern side of the Estonian islands of Hiiumaa and Saaremaa (samples GFE‐5 and 6), where oceanographic currents from both the Gulf of Finland, the Bay of Riga, and the Baltic Proper converge, and also in the Västervik area on the Swedish southeast coast (sample SBA‐4) (samples marked in Figure 2), which harbors the highest amount of diversity of all samples collected along the Baltic Sea coasts. Establishment of MPAs in these areas would probably be very beneficial to the long‐term sustainability of the Baltic *I. balthica* population in general.

Finally, as significant warming and decrease in salinity are expected in the Baltic Sea before the end of this century, local extinctions of isopod populations throughout the area, especially near current range margins, are to be expected (Kotta et al., 2019). These could in turn have immediate negative consequences for ecosystem functions as isopods are important as food for, for example, local populations of herring and perch. While local extinctions might be unavoidable at the most northern areas, populations further south could be supported by influx of genetic material, which might contain low‐salinity tolerant traits, from the north. A potential management strategy to minimize the detrimental effects of climate change would thus be to facilitate dispersal southward along the salinity gradient by protecting suitable habitats (continuous *Fucus* belts), especially along the Swedish Bothnian Sea and northern Baltic Sea coasts, as it may otherwise be difficult for low‐salinity adapted genotypes to track the receding salinity gradient (Kotta et al., 2019). This is especially important as our data show that the Swedish Bothnian Sea coast is genetically isolated. There is currently a lack of designated MPAs in this particular area aiming to protect *Fucus* spp., an overview of which seems motivated to maintain stepping‐stone possibilities for north to south gene flow in *I. balthica*. It might also be considered to physically translocate northern populations to more southerly areas. While this type of manipulation is controversial, it might prove necessary under rapidly decreasing salinities in order to sustain more southern Baltic Sea populations of isopods. Surface salinity has been decreasing since 1980 and may reach 3 psu in large parts of the Baltic Proper before the end of the century (Meier, Andersson, et al., 2012b; Meier, Kjellström, & Graham, 2006). However, it is still unclear exactly how the interactions of all projected environmental changes will affect species distributions within the next hundred years, so consequently it will be important to establish genetic monitoring, in order to be able to give early warnings for loss of genetic diversity in of both *Fucus* spp. and *I. balthica*.

## Data Availability

All raw sequencing data were submitted to the NCBI Short Read Archive, BioProject PRJNA551577. Vcf files containing all genotype calls were submitted to the Dryad online repository (https://doi.org/10.5061/dryad.xksn02vbk) (De Wit et al., 2019). The unpublished genome sequence is available upon request at http://albiorix.bioenv.gu.se/Idotea_balthica.html.
