# Supplementary material for: Spatial genetic structure in a crustacean herbivore highlights the need for local considerations in Baltic Sea biodiversity management
Source: Evol Appl. 2020 Feb 5;13(5):974–90. doi: 10.1111/eva.12914 (PMC7232771; doi:10.1111/eva.12914)
Supplement: Supplementary file 1 [file EVA-13-974-s001.pdf]

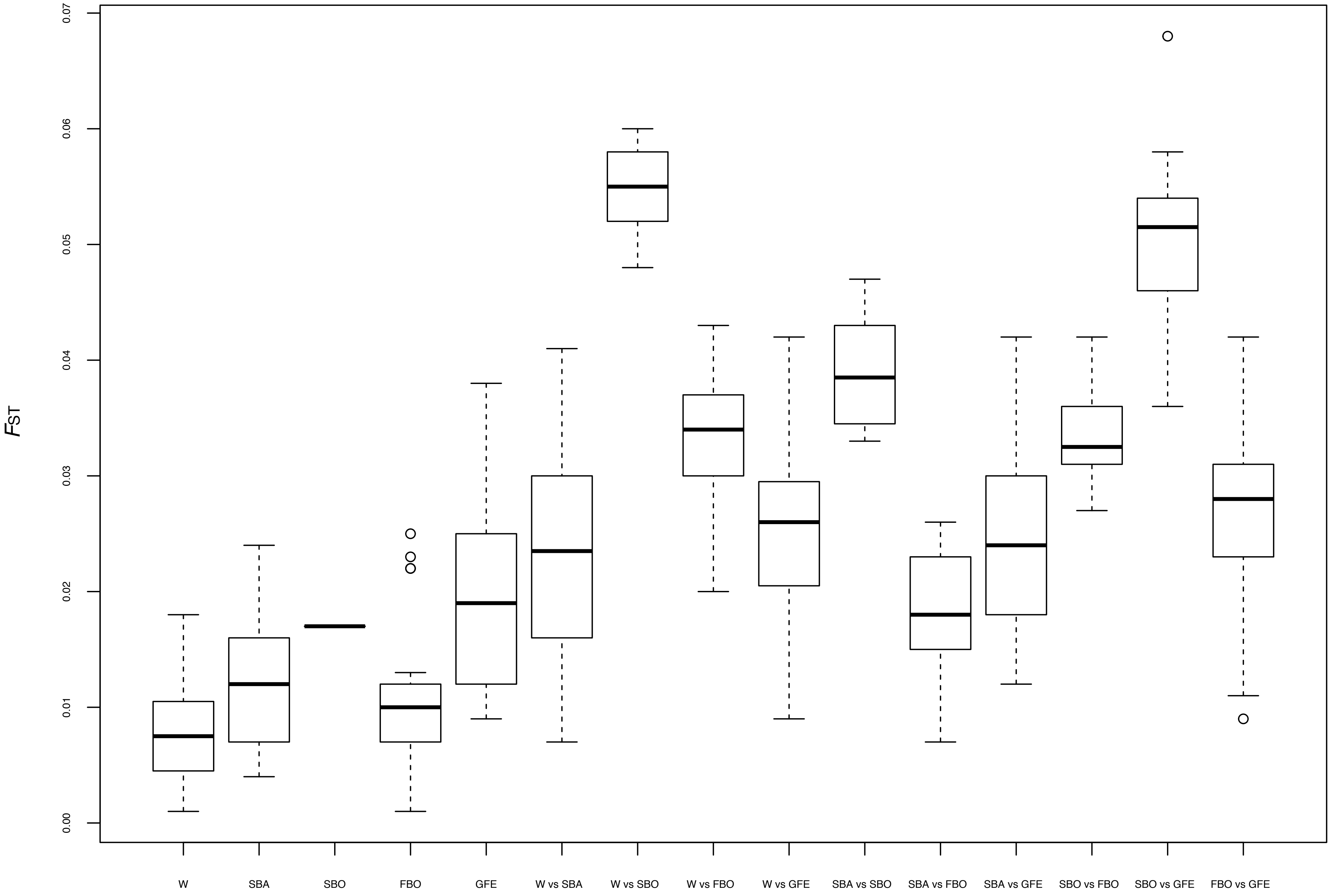

Supplementary Figure 1. Boxplots of F_{ST} distributions within and among regions of the study (W -West; SBA - Swedish Baltic Sea; SBO - Swedish Bothnian Sea; FBO - Finnish Bothnian Sea; GFE - Gulf of Finland and Estonia).
