# Supplementary material for: Spatial genetic structure in a crustacean herbivore highlights the need for local considerations in Baltic Sea biodiversity management
Source: Evol Appl. 2020 Feb 5;13(5):974–90. doi: 10.1111/eva.12914 (PMC7232771; doi:10.1111/eva.12914)
Supplement: Supplementary file 3 [file EVA-13-974-s003.pdf]

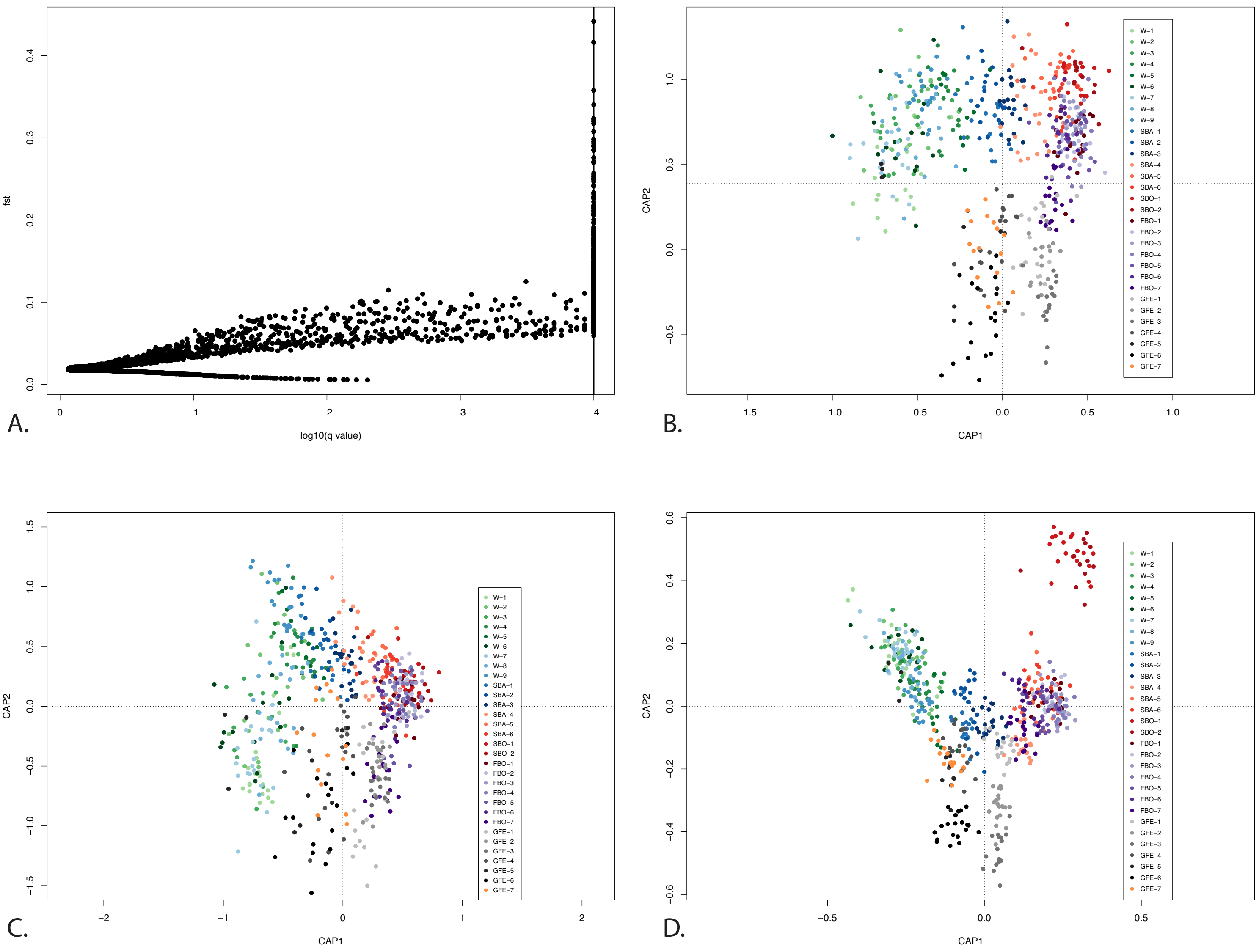

Supplementary Figure 3. Results from outlier analysis: A. BayeScan output: F_{ST} values plotted against FDR-corrected p-values (vertical line) = 10e-4; B. CAP-plot of 487 F_{ST} outliers identifed by BayeScan (Foll & Gaggiotti 2008); C. CAP-plot of 170 F_{ST} outliers identifed by OutFLANK (Whitlock & Lotterhos 2015); D. CAP-plot of non-outliers only.
