# Supplementary figures and images for: Spatial genetic structure in a crustacean herbivore highlights the need for local considerations in Baltic Sea biodiversity management
Source: Evol Appl. 2020 Feb 5;13(5):974–90. doi: 10.1111/eva.12914 (PMC7232771; doi:10.1111/eva.12914)

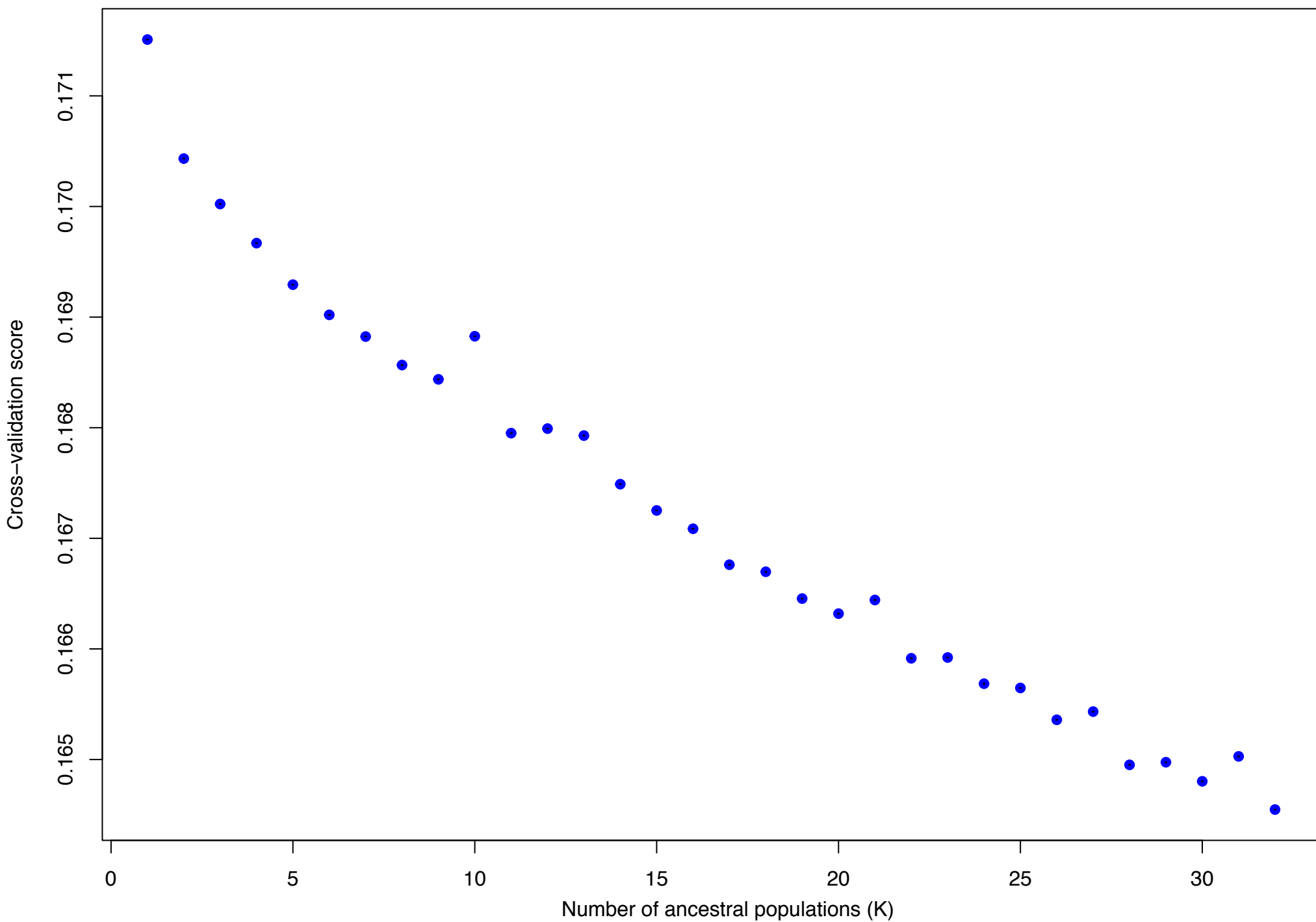

**Supplementary Figure 5.** Cross-validation scores of admixture output, with K ranging from 2 to 32.

Supplement: Supplementary file 5 [file EVA-13-974-s005.pdf]
