# Supplementary material for: Spatial genetic structure in a crustacean herbivore highlights the need for local considerations in Baltic Sea biodiversity management
Source: Evol Appl. 2020 Feb 5;13(5):974–90. doi: 10.1111/eva.12914 (PMC7232771; doi:10.1111/eva.12914)
Supplement: Supplementary file 4 [file EVA-13-974-s004.pdf]

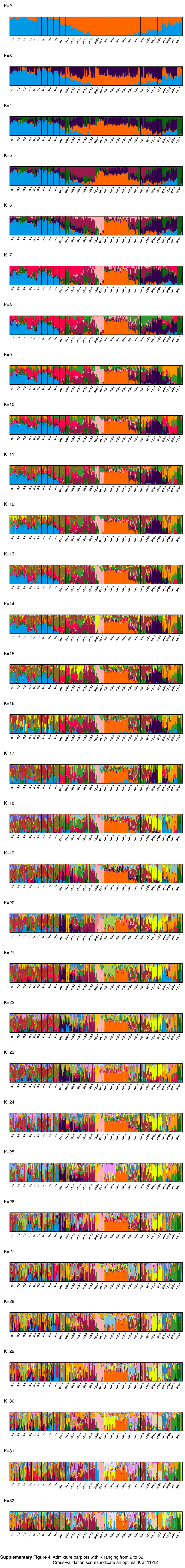

Supplementary Figure 4. Admixture barplots with K ranging from 2 to 32.
Cross-validation scores indicate an optimal K at 11-12
